# Siesta and Risk for Ischemic Stroke: Results from a Case-Control Study

**DOI:** 10.3390/medicina56050222

**Published:** 2020-05-07

**Authors:** Yousef Mohammad

**Affiliations:** Associate Professor of Neurology, Department of Internal Medicine, King Saud University, P.O. Box 7805, 11472 Riyadh, Saudi Arabia; ymohammad@ksu.edu.sa; Tel.: +966-5-693-81218

**Keywords:** siesta, afternoon nap, ischemic stroke, coronary artery disease

## Abstract

*Background and objectives*: Siesta, which is a short afternoon nap, is a habit that is commonly practiced in the Mediterranean and tropical areas. Data on the association between siesta and coronary artery disease has been conflicting. A protective effect has been demonstrated in the countries that commonly practice siesta, but a harmful effect has been observed in the countries that infrequently practice the siesta habit. Information on the association between siesta and ischemic stroke has been, however, lacking. Hence, the purpose of our study was to determine the effect of siesta on ischemic stroke. *Materials and Methods*: This was a case-control study, conducted on the patients with acute ischemic stroke who came for their first follow-up visit to the neurology clinic. Controls were randomly selected from the patients visiting the neurology clinic on the same day as the patients with ischemic stroke. In addition to basic demographics and the occurrence of established stroke risk factors, information about siesta practice was also collected from both groups. A multivariate logistic regression analysis was utilized to determine the relationship between siesta practice and ischemic stroke. *Results*: A total of 206 patients were recruited from the neurology clinic of King Khalid university hospital; of which only 194 subjects were included in the analysis (98 ischemic stroke cases and 96 controls). The mean age of the participants was 59.68 ± 13.75 years and 98 (50.52%) were male. Interestingly, 43% of the whole study cohort practiced regular siesta. However, when compared to the stroke population, the control group practiced siesta more frequently (30% vs. 56%). In a multivariate logistic regression analysis, hypertension, diabetes mellitus, excess body weight (body mass index > 25 kg/m^2^) and dyslipidemia were found to increase the risk of ischemic stroke (OR 2.12, 95% CI: 1.02–4.66, *p* = 0.005; OR 2.72, 95% CI: 1.94–4.88, *p* = 0.014; OR 2.94, 95% CI: 1.5164–5.7121 *p* = 0.0014; OR 3.27, 95% CI: 2.42–5.199, *p* ≤ 0.001, respectively). On the contrary, the practice of regular siesta lowered the risk of ischemic stroke (OR 0.58, 95% CI: 0.3551–0.9526, *p* = 0.031). *Conclusions*: Siesta was associated with a reduced risk for the occurrence of ischemic stroke. Large prospective longitudinal studies should be conducted to verify the protective effect of siesta on stroke.

## 1. Introduction

Worldwide, stroke is the second leading cause of mortality [1] and the third most common cause of long-term disability in adults [2]. Despite recent advances, the effects of acute ischemic stroke treatment [3,4,5] remain limited, and stroke prevention remains the most crucial factor in its management [6]. Multiple risk factors of ischemic stroke have been identified (e.g., hypertension, diabetes mellitus, dyslipidemia, cigarette smoking and obesity) [7], and the control of these risk factors has resulted in a large reduction in the incidence and prevalence of ischemic stroke [8]. Hence, the quest for hidden risk factors has been strongly recommended by the various esteemed stroke organizations.

Stroke onset has been observed to follow a circadian rhythm, occurring mainly in the early morning hours after waking up from night sleep [9,10]. So, research has been intensively directed toward exploring the relationships among the various sleep disorders and stroke. Indeed, certain sleep disorders have already been established as important risk factors for the occurrence of ischemic stroke. For example, obstructive sleep apnea, which is highly prevalent among the general population and is a treatable condition, has been shown in multiple epidemiologic studies to significantly increase the risk of ischemic stroke [11,12]. Moreover, it has been associated with worse outcomes [13]. At the same time, it has been demonstrated, from multiple investigations done across nations, that sleeping longer than 8 h per night increases the incidence of, and mortality rate from, stroke [14,15,16]. Similarly, excessive day time sleepiness has been repeatedly demonstrated to independently increase the risk of stroke [15,17,18].

Siesta, which is a short afternoon nap, is a habit that is commonly practiced in Mediterranean and tropical areas. For instance, 60%–80% of the adult populations living in Nigeria, Mexico and Ecuador frequently take a siesta [19]. A low incidence of coronary artery diseases has been observed in the regions where siesta is commonly practiced [20]. However, initial data on the association between siesta and coronary artery disease is conflicting. For example, studies done in Greece showed that siesta is protective against coronary artery disease [21,22,23] (Table 1), whereas reports from Israel and Costa Rica indicated that siesta increases the incidence and mortality rate of coronary artery disease [24,25,26] (Table 1). Indeed, multiple recent meta-analyses and large longitudinal population-based studies have demonstrated an increased risk of coronary artery disease and mortality with siesta [27,28,29]. Data on the association between siesta and ischemic stroke is, however, lacking. Hence, we conducted this study to determine the relationship between siesta and ischemic stroke.

## 2. Methods

### 2.1. Study Design and Subjects

This case-control study was conducted at the neurology clinic of King Khalid university hospital (Riyadh, Saudi Arabia). The participants were consecutive patients suffering from acute ischemic stroke who came for their first follow-up visit at the neurology clinic; in all patients the first follow-up visit occurred within two weeks after discharge from the hospital. All occurrences of ischemic stroke were confirmed by diffusion weighted sequence magnetic resonance imaging of the brain, which had been performed during the patients’ most recent hospital admission.

Controls, matched for age (±5 years) and gender, were randomly selected from the patients visiting the neurology clinic on the same day as the ischemic stroke patients, and were excluded if they had any history of stroke, coronary artery disease, or peripheral artery disease. The usual neurological disorders seen in the outpatient neurology clinic include strokes, headaches, epilepsy, dementia, movement disorders, radicular pain, multiple sclerosis, and traumatic neurological injuries. The patients with ischemic stroke and the controls, who agreed to participate in the study, were interviewed by a stroke specialist who was blinded to the purpose of the study. They were asked to answer a list of questions related to demographics, health status, and siesta practice prior to the ischemic stroke.

Prior to the study launch, approval of the study protocol was obtained from the institutional review board of King Khalid university hospital. Similarly, verbal consent was procured from the patient or their guardian prior to data collection. Ethical code: E-18-4376, approved date: October 15.

### 2.2. Study Variables

Siesta was defined as an afternoon nap, and siesta practice was divided into two frequency categories: occasional siesta (< 2 naps per week) and regular siesta (≥ 2 naps per week).

Demographic and baseline variables collected included age, gender, body mass index, smoking status, and history of diabetes mellitus, hypertension, dyslipidemia and atrial fibrillation.

### 2.3. Statistical Analysis

SPSS Statistics Version 26 software, which is the most recent version of the software package (SPSS 12; College Station, TX, USA), was employed for all statistical analyses. The student t-test was utilized to assess whether there were any differences between the ischemic stroke group and the control group for all continuous variables, and the results are presented as the mean plus or minus the standard deviation. The distribution of all categorical variables, in the two groups, was compared through the application of the Chi-square test. A *p* value of < 0.05 was determined to represent a significant difference for all statistical analyses. The multivariate logistic regression analysis was deemed essential because many of the risk factors for ischemic stroke are intercorrelated, and this analysis was utilized to determine the associations between the various clinical features, including siesta practice, and ischemic stroke.

## 3. Results

Between October 2018 and August 2019, 206 patients were recruited from the neurology clinic of King Khalid university hospital: 103 ischemic stroke cases and 103 controls. However, only 194 subjects were included in the analysis (98 stroke cases and 96 controls), because 5 stroke cases and 7 controls were excluded due to missing data for any of the requested variables. The mean age was 59.68 years, and 98 (50.52%) of the participants were male; and there were no differences between the two groups as these were matching variables (Table 2). The neurological disorders present in the control group included headache (28%), epilepsy (26%), radicular pain (14%), dementia (9%), movement disorders (9%) and others (14%).

The study population was, in general, overweight (mean body mass index [BMI] of 27.63 kg/m^2^), and a significant proportion were hypertensive or diabetic; 49% and 44% respectively. Furthermore, around one quarter had hyperlipidemia or were smokers. However, only a small fraction had atrial fibrillation (Table 2).

As predicted, the ischemic stroke group had greater prevalence of hypertension, diabetes mellitus and dyslipidemia (Table 2). However, no differences were detected between the two groups with respect to the prevalence of smoking and atrial fibrillation.

Intriguingly, more than one third (43%) of the whole study population took a regular afternoon nap. However, there was a difference in the frequency of siesta between the ischemic stroke and control cases. In fact, when compared with the stroke population, the control group practiced regular siesta more frequently: 30% and 56%, respectively (Table 2).

In the multivariate logistic regression analysis model, both hypertension and diabetes were found to increase the risk of ischemic stroke (OR of 2.12 and 2.72, respectively). Similarly, both extra body weight (BMI > 25) and dyslipidemia were associated with an increased risk of ischemic stroke (OR of 2.94 and 3.27, respectively). On the other hand, regular siesta was inversely associated with stroke (OR 0.58) (Table 3). 

## 4. Discussion

The results of our study revealed an inverse relationship between regular siesta and ischemic stroke. Data in the literature, pertaining to the relationship between siesta and coronary artery disease, is abundant. However, our report is the first to address the link between siesta and ischemic stroke. 

Our findings are consistent with the results of multiple reports that assessed the role of siesta in coronary artery disease. For example, in two separate case-control studies done in Greece, both Trichopoulos et al. and Kalandidi et al. [21,22] showed a decreased risk of coronary artery disease (CAD) in the subjects who practiced siesta. Similarly, in a prospective longitudinal study that was also done in Greece, Naska et al. [23] demonstrated a protective effect of siesta against CAD. Reports from China also showed that siesta lowers the risk of CAD [30]. In fact, this is also consistent with the low prevalence of CAD in the areas that commonly practice siesta, such as Mediterranean countries, Latin American countries, and the tropical regions [20]. Siesta delivers its protection against ischemic stroke and CAD by being a natural stress relief and stress coping technique [31,32]. It has also been postulated that the afternoon hours are associated with reduced platelet aggregation and lower coronary vasoconstriction propensity [33], which is another explanation for the lower rate of ischemic stroke and CAD when siesta is practiced. 

Contrary to our results, however, other reports showed that siesta increases the risk of CAD and mortality. For instance, in a case-control study done in Costa Rica, Campos et al. showed that taking a daily siesta is associated with a higher risk of CAD [24]. Similarly, in two prospective longitudinal studies done in Israel, both Bursztyn et al. and Burazeri et al. [25,26] associated an increased risk of CAD and mortality with siesta practice. The morning waking hours, when activities begin, are accompanied with activation of the sympathetic adrenergic network, and subsequent surges in systolic blood pressure and heart rate, which explain the rise in the occurrence of ischemic stroke and CAD during this period [34,35]. Similar hemodynamic changes were predicted to occur in the early hours after waking up from siesta comparable to those that occur after waking up from nocturnal sleep. Hence, the authors, who implicated siesta as the cause of CAD, exploited this hypothesis to support the merit of their results. However, it has been demonstrated that the hemodynamic changes that occur after siesta are different from those that occur after nocturnal sleep. For instance, Mulcahy et al. reported smaller surges in heart rate and systolic blood pressure after siesta when compared to the surge that occurs after nocturnal sleep [33]. Likewise, Bursztyn et al. found lower rises in heart rate and double-product (systolic blood pressure multiplied by the heart rate) in the interval that follows siesta [36]. In other words, after siesta, there was a lower demand for cardiac oxygen consumption and, thus, a lower tendency for ischemia to occur. It is also intriguing to notice that studies done in countries that commonly practice siesta showed a beneficial effect against CAD, whereas studies done in areas that infrequently practice siesta demonstrated a harmful effect. This observation suggests that the effect of siesta on CAD might be ethnicity specific. In other words, siesta might be hardwired into the genes of only the populations who frequently practice siesta. In fact, a gene that regulates siesta has already been discovered in insects that are dormant in the afternoon [37]. 

Our study had a few limitations. First, socioeconomic status is known to affect the incidence of ischemic stroke, and might have had a confounding effect on the results. This important variable was not addressed in our study and must be examined in future research. Second, a few reports have demonstrated that prolonged siesta plays a factor in the occurrence of stroke and CAD. In our study, however, data on the duration of siesta were not collected. Third, the presence of obstructive sleep apnea, which is one of the most common sleep disorders among the adult population and a major risk factor for ischemic stroke and CAD, was not determined in our study. Therefore, in our analysis, we did not adjust for this potential confounding variable. Fourth, our study only included two siesta practice categories. However, a more comprehensive list of siesta practice frequencies, such as < 1 nap/week, 1–4 naps/week, 5–6 naps/week, or daily naps, would definitely be more informative. Fifth, dietary practices and physical exercise, which have been shown to affect the incidence and prevalence of ischemic stroke and CAD, were not addressed in our study.

In conclusion, siesta is common in the tropical and Mediterranean areas. Epidemiological studies have demonstrated a lower prevalence of CAD and ischemic stroke in these areas. Multiple case-control and prospective longitudinal studies conducted in the areas that regularly practice siesta showed a protective effect on CAD from siesta. Our study showed that the beneficial effects of siesta also extend to ischemic stroke. It is strongly advised that larger prospective studies, including healthy people as a control group and incorporating the siesta duration and all other confounding ischemic stroke risk factors, should be conducted to verify the effect of siesta on ischemic stroke. In the meantime, definitive advice or opinions on siesta practice should not be provided.

## Figures and Tables

**Table 1:** Summary of the studies that have assessed the effect of siesta on CAD.

Study	Country	Sample Size	Siesta Duration	Siesta Frequency	CAD Occurrence
Trichopoulos	Greece	187	30 min	Undocumented	Reduced
Kalandidi	Greece	899	30 min	Undocumented	Reduced
Naska	Greece	23,681	≥30 min	≥3 times a week	Reduced
Campos	Costa Rica	1027	1–2 h	Daily	Increased
Bursztyn	Israel	455	Unknown	Undocumented	Increased
Burazeri	Israel	1859	≥2 h	Regular	Increased

Siesta: afternoon nap; CAD: coronary artery disease.

**Table 2:** Demographics and clinical characteristics.

Variables	Total N (%)	Stroke	Controls	*p* Value
		N (%)	N (%)	
Age (Mean ± SD)	59.68 ± 13.75	59.84 ± 13.78	59.51 ± 13.61	0.67
≤49	50 (25.77)	24 (24.5)	26 (27.08)	
50–59	48 (24.74)	25 (25.5)	23 (23.96)	
60–69	44 (22.68)	22 (22.4)	22 (22.92)	
≥70	52 (26.80)	27 (27.6)	25 (26.04)	
Gender				
Male	98 (50.52)	49 (50)	49 (51.04)	0.88
Female	96 (49.48)	49 (50)	47 (48.96)	
BMI (Mean ± SD)	27.78 ± 4.70	28.97 ± 4.67	26.58 ± 4.71	0.08
<25	52 (26.8)	21 (21.42)	31 (32.29)	
25–30	87 (44.85)	39 (39.79)	48 (50.00)	
>30	55 (28.35)	38 (38.77)	17 (17.71)	
Hypertension				
Yes	95 (48.97)	67 (68.37)	28 (29.17)	<0.0001 *
No	99 (51.03)	31 (31.63)	68 (70.83)	
Diabetes mellitus				
Yes	85 (43.81)	61 (62.24)	24 (25.00)	<0.0001 *
No	109 (56.19)	37 (37.76)	72 (75.00)	
Smoking				
Yes	41 (21.13)	22 (22.45)	19 (19.79)	=0.65
No	153 (78.87)	76 (77.55)	77 (80.21)	
Atrial Fibrillation				
Yes	17 (8.76)	9 (9.18)	8 (8.33)	=0.83
No	177 (91.24)	89 (90.82)	88 (91.67)	
Siesta				
Regular Siesta *	83 (42.79)	29 (29.59)	54 (56.25)	=0.0002 *
Occasional siesta **	111 (57.22)	69 (70.41)	42 (43.75)	
Dyslipidemia				
Yes	61 (31.44)	38 (38.78)	23 (23.96)	=0.02 *
No	133 (68.56)	60 (61.22)	73 (76.04)	
Total	194 (100)	98 (50.5)	96 (49.5)	

Regular siesta *: ≥ 2/week; Occasional siesta **: < 2/week. *p* value < 0.05 is statistically significant.

**Table 3:** Stroke. Multinomial logistic regression analysis for siesta and established risk factors.

Risk Factor	OR	95% CI	*p*
Hypertension			
Yes	2.1	1.02–4.66	=0.005
No	1	1	
Diabetes mellitus			
Yes	2.72	1.94–4.88	=0.014
No	1	1	
Smoking			
Yes	1.25	0.98–2.41	=0.08
No	1	1	
Atrial fibrillation			
Yes	1.14	0.74–3.01	=0.51
No	1	1	
BMI			
Excess body weight	2.94	1.5164–5.7121	=0.0014
Normal weight	1	1	
Siesta			
Regular siesta *	0.58	0.3551–0.9526	=0.031
Occasional siesta **	1	1	
Dyslipidemia			
Yes	3.27	2.42–5.19	<0.001
No	1	1	

Regular siesta *: ≥ 2/week; Occasional siesta **: < 2/week. OR: Odds ratio; 95% CI: 95% confidence interval. *p* value < 0.05 is statistically significant.
